# Seasonal changes in physical capacities of basketball players according to competitive levels and individual responses

**DOI:** 10.1371/journal.pone.0230558

**Published:** 2020-03-19

**Authors:** Davide Ferioli, Andrea Bosio, James Zois, Antonio La Torre, Ermanno Rampinini

**Affiliations:** 1 Department of Biomedical Sciences for Health, Università degli Studi di Milano, Milano, Italy; 2 Human Performance Laboratory, MAPEI Sport Research Centre, Olgiate Olona, Varese, Italy; 3 Institute of Health and Sport, College of Sport and Exercise Science, Victoria University, Melbourne, Victoria, Australia; 4 IRCSS, Istituto Ortopedico Galeazzi, Milano, Italy; University of Cassino e Lazio Meridionale, ITALY

## Abstract

**Purpose:**

The aim of this study was to quantify changes in physical capacities of thirty-eight basketball players selected from different teams, as well as from varying competitive levels (i.e. Division I, Division II and Division III) during the preparation and in-season periods.

**Methods:**

Pre (T1) and post (T2) preparation period and during regular season (T3), the players completed a Yo-Yo Intermittent Recovery test—level 1. Following a 3 to 8 days-break, players performed a 6-min continuous running test (Mognoni’s test), a counter-movement jump test and a 5-min high-intensity intermittent running test.

**Results:**

Blood lactate concentration measured after the Mognoni’s test was significantly reduced from T1 to T2, and from T2 to T3 (*P*<0.001, ƞ^2^ = 0.424). The distance covered during the Yo-Yo Intermittent Recovery test was significantly increased only from T1 to T2 in Division II and III (*P*<0.001, ƞ^2^ = 0.789). Similarly, the physiological responses to high-intensity intermittent running test were improved only from T1 to T2 (all *P*<0.001, ƞ^2^ = 0.495 to 0.652). Despite significant changes observed in running tests from T1 to T2, at individual level 35–55% of players did not show a very likely improvement. Relative peak power produced during vertical jumps at T3 by Division I players was increased compared to T1 (ANOVA interaction, *P* = 0.037, ƞ^2^ = 0.134).

**Conclusions:**

The main improvements in physical capacities occurred during the preparation period, when the aerobic fitness and the ability to sustain high-intensity intermittent efforts were moderately-to-largely improved. However, it appears that the preparation period does not consistently impact on vertical jump variables. Aerobic fitness and force/power production during vertical jumps appear to improve across the competitive season (slightly-to-moderately). Physical tests should be used to identify weaknesses in physical performance of players and to monitor their fatigue status, with the aim to develop individualized training programs.

## Introduction

Basketball is an intermittent team sport characterized by alternating low- and high-intensity phases, often requiring a variety of specific technical skills, frequent changes of direction and jumps [1, 2]. The aerobic and anaerobic mechanisms are heavily activated to provide energy during basketball practice [3]. Accordingly, the ability to sustain high-intensity intermittent efforts and to produce strength and power are important physical determinants during basketball competitions [3].

The assessment of players’ physical fitness across an entire basketball season indicates the effectiveness of conditioning programs and permits quantification of changes in fitness status of players across various phases of the season [4]. The greatest improvement in an athlete’ physical fitness usually occurs during the preparation period, when players begin performing physical activity after a prolonged period of complete, or nearly complete rest [4–6]. During the competitive phase of the season strength and conditioning programs aim to maintain players’ physical fitness, although realistically, fitness may slightly increase or decrease [4, 7]. In addition, different individual responses to basketball practice might be expected among players belonging to the same team [8] for several reasons such as playing time, injuries and fatigue status. As such, strength and conditioning coaches should take into consideration the fitness status of their players in developing individualized training sessions or tapering strategies.

Despite several studies investigating the seasonal changes in physical fitness of junior and collegiate (National Collegiate Athletic Association) basketball players [4, 9–15], only few studies have investigated these trends in adult male professional basketballers [5–8, 16]. Gonzalez et al. [8] investigated performance changes among 7 NBA basketball players from the beginning to the end of the regular season, reporting improvements in lower limb power produced during squat exercise and repeated vertical jumps. Furthermore, starters maintained their body mass and percentage of body fat during the regular season. Aoki et al. [16] reported small-to-large improvements in vertical jumping performance and moderate-to-large greater distances covered during the Yo-Yo Intermittent recovery test (Yo-YoIR1) among 9 professional players during the preparation period (i.e. 4^th^ week from the beginning) and after 3 weeks from the beginning of the Brazilian regular season. Similarly, the preparation period has been shown to be effective in enhancing the ability to sustain high-intensity intermittent efforts and repeated changes of direction, but to be less effective in improving the aerobic fitness and jumping ability of professional basketball players competing in Italian tournaments [5, 6].

These studies provide some preliminary indications pertaining to changes in physical capacities over the preparation period in professional adult male basketball players. However, it should be noted that most of these studies involved a limited number of players from the same team, thus making generalization of the findings difficult. Additionally, only two studies have assessed the physical capacities of professional adult players across different phases of the entire season (i.e. preparation period and in-season period) [7, 16]. The paucity of scientific data may be due to the difficulty associated with conducting longitudinal research in professional athletes across an entire basketball season. Accordingly, further research needs to be conducted to advance the knowledge on this important topic.

Previous studies reported changes in physical fitness of basketball players to be affected by the competitive level of play [4–7, 17]. Thus, a thorough understanding of seasonal fitness variations at varying playing standards might highlight useful information for physical preparation staff and coaches alike. Accordingly, providing indicative data of seasonal changes in physical capacities of basketball players according to their competitive level might assist strength and conditioning coaches and practitioners to better understand the effectiveness of the developed training programs across the different phases of the season. Furthermore, the data may provide a clearer interpretation of the fitness status of the players during the season, when no previous physical assessment information are available (e.g. recruiters). Therefore, the aim of this study was to quantify the changes in physical capacities of basketball players selected from different teams, as well as from varying competitive levels, during preparation and in-season periods.

## Materials and methods

### Subjects

Thirty-eight male basketball players competing in the Italian Serie A (Division I, n = 13, age: 27 ± 6 years, stature: 202 ± 9 cm), Serie A2 (Division II, n = 12, age: 24 ± 4 years, stature: 198 ± 8 cm) and Serie B (Division III, n = 13, age: 24 ± 5 years, stature: 193 ± 8 cm) were recruited from a total of 7 basketball teams (i.e. 2 or 3 teams for each division). Division I and Division II athletes trained 6 to 10 times a week, while Division III players performed 4 to 7 training sessions per week. In all divisions the athletes performed two strength training sessions in addition to a conditioning session per week. Training sessions lasted 60–120 min, including warm-up and excluding cool down and/or stretching exercises. Division I teams played 1–2 games per week, while Division II and III teams completed one game per week. All basketball players included in this study performed more than 80% of the team training sessions and were free of injury at least in the 6 months before the testing period [18]. After verbal and written explanation of the experimental design and potential risks and benefits of the study, written informed consent was signed by all players. The study was approved by the Independent Institutional Review Board of MAPEI Sport Research Centre in accordance with the Helsinki Declaration.

#### Anthropometric characteristics

Stature (stadiometer Wall Mounted, mod206 Seca, Birmingham UK), body mass (portable scale mod762 Seca, Birmingham UK) and body fat (Harpenden skinfold caliper, Lanzoni srl, Bologna, Italy) percentage were determined prior to commencement of the physical testing sessions. The estimation of the body density was determined through the equation eight as described by Jackson and Pollock [19] using skin-fold (i.e. chest, abdomen and thigh) and circumference (i.e. forearm and waist) measures. The estimated body density was then transformed to body fat percentage using the Siri’s equation [20].

### Design and methodology

An observational study was used to assess the seasonal fitness variations of basketball players of different competitive levels. Players were assessed 3 times during the entire basketball season 2015–16 or 2016–17: the first week of the preparation period (T1, mid-August); within the first 2 weeks from the start of the competitive season (T2, mid-October); and during the competitive phase of the season (T3), at least 9 weeks after T2 (i.e. from end-January to early-March over a period of 6 weeks). At all-time points (i.e. T1, T2 and T3) testing sessions were completed in the morning (from 9.30 am to 12.30 pm) on two separate test days. On day 1 the players underwent a Yo-YoIR1, on day 2 they performed a physical test session consisting of a continuous running test (Mognoni’s test), followed by a counter-movement jump (CMJ) test and by a high-intensity intermittent running test (HIT). The second test day was carried out between 3 to 8 days following the Yo-YoIR1. Due to restrictions made by technical coaches the Division I athletes did not carry out the Yo-YoIR1. To avoid potential confounding effects of prior exercise fatigue on the outcome variables no training sessions were performed the day preceding the assessments. In addition, no stretching exercises were allowed prior to the tests. These procedures have been previously carried out to assess professional, semi-professional and amateur players in basketball [21]. All players were familiar with the tests performed in the present study.

#### Yo-Yo intermittent recovery test—Level 1

Yo-YoIR1 consisted of 20-m shuttle runs performed at increasing velocities (beginning speed of 10 km∙h^-1^) with 10 s of active recovery (consisting of 2x5-m of jogging) between runs until exhaustion [22, 23]. The test concluded when participants failed to complete the distance in time twice (objective evaluation) or due to volitional fatigue (subjective evaluation). The total distance covered during Yo-YoIR1 was considered as the test “score” [23]. Heart rate was continuously monitored using Team^2^ Pro System (Polar, Kempele, Finland) and all the athletes achieved at least the 90% of the predicted maximal heart rate, estimated as 220—age [24]. Reliability and validity of this test have been previously reported in basketball literature [22, 23].

#### Continuous running test (Mognoni’s)

The Mognoni’s test consisted of a 6-min continuous run at a constant speed of 13.5 km∙h^-1^ on a motorized treadmill (HP Cosmos, Nussdorf—Traunstein, Germany) [21, 25, 26]. Capillary blood lactate concentration (MOG_[La-]_) was measured from the earlobe immediately after the completion of the test using a portable amperometric microvolume lactate analyser (Lactate Plus, Nova Biomedical, Waltham, MA, USA). Heart rate was continuously monitored using Team^2^ Pro System (Polar, Kempele, Finland) and the mean heart rate (MOG_HR_) of the last minute of running was considered for analysis. Athletes were instructed to abstain from any kind of warm-up prior to the test to avoid potential confounding effects on the physiological responses to the Mognoni’s test. This test provides a reliable, simple and feasible method to assess aerobic fitness [21, 25], which is considered important for recovery during high-intensity intermittent exercise [27].

#### Counter-movement jump test

One minute prior to CMJ testing athletes carried out two submaximal CMJs. The CMJ test was performed using a portable force platform (Quattro Jump, Kistler, Winterthur, Switzerland) and its application software (Version 1.1.1.4) 10 minutes post the Mognoni’s test. Each athlete performed 5 bilateral single CMJs, separated by 30 s of passive rest, from a standing position with hands placed on the hips to minimize any influence of the arms. Players were instructed to perform a quick downward movement reaching about 90° knee flexion, promptly followed by a fast-upward movement, with the aim to jump as high as possible. During the concentric phase of each CMJ peak power output (PPO), peak force (PF) and jump height (CMJ_h_) were recorded. The average of the best 3 values was used for analysis. Reliability and validity of this test have been widely reported in basketball literature [5, 21, 28].

#### High-intensity intermittent test

The HIT protocol [6, 21], comprising of 10×10 s shuttle runs over a 25+25 m course with a 180° change of direction and 20 s of passive recovery between each bout, was performed 10 minutes after the end of the CMJ test. The players were required to run at 18 km∙h^-1^, following a sequence of audio signals. Immediately after the HIT protocol, a 100 μL capillary blood sample was drawn from an earlobe into a heparinised capillary tube and analysed for blood hydrogen ion concentration (HIT_[H+]_) and bicarbonate concentration (HIT_[HCO3-]_) using a calibrated blood-gas analyser (GEM Premier 3000, Instrumentation Laboratory, Milan, Italy) with an Intelligent Quality Management System cartridge and for blood lactate concentration (HIT_[La-]_) using a portable amperometric microvolume lactate analyser (Lactate Plus, Nova Biomedical, Waltham, MA, USA). Heart rate was continuously monitored using Team^2^ Pro System (Polar, Kempele, Finland) and the mean heart rate of the test (HIT_HR_) was considered for statistical analysis. The HIT represents a valid and reliable tool to investigate the ability to sustain high-intensity intermittent efforts in basketball in a submaximal and systematic way [6, 21].

### Statistical analysis

The participants’ descriptive results are reported as means ± standard deviations (SD). The assumption of normality was verified by the Kolmogorov-Smirnov test for each variable. A series of two-way repeated-measures ANOVA (Divisions × time) was utilized to assess differences. The independent variables included two factors: a) three levels for Divisions (Division I, II and III) and b) three levels for time (T1, T2 and T3). However, the Yo-YoIR1 scores were analysed using a 2×3 repeated-measures ANOVA as only Division II and III players performed the Yo-YOIR1. Partial eta-squared (ƞ^2^) was used as a measure of effect size and values were classified as follows: ƞ^2^<0.04, no effect; 0.04 <ƞ^2^<0.25, minimum effect; 0.25 <ƞ^2^<0.64, moderate effect; ƞ^2^>0.64, strong effect [29]. When significant *F* values were found, Bonferroni post hoc tests were used and both percentage of change in mean and Cohen’s d effect size (ES) [30] with 95% confidence intervals were calculated. ESs were considered as follows: <0.20, trivial; 0.20–0.59, small; 0.60–1.19, moderate; 1.20–1.99, large; and 2.00–4.00, very large [31]. Statistical significance was set at *P* <0.05. For each tests variables individual responders (*very likely* change, probability of a positive or negative change >90%) were determined according to Hopkins [32] as previously carried out in basketball [33, 34]. The typical error of measurement (test-retest reliability) expressed as coefficient of variation (CV) of all the tests variables has been described previously [21, 23], resulting as follows: body mass, 0.7%; body fat percentage, 3.4%; MOG_[La-]_, 8.0%; MOG_HR_, 0.8%; HIT_[La-]_, 12.4%; HIT_[H+]_, 5.3%; HIT_[HCO3-]_, 7.2%; HIT_HR_, 2.3%; Yo-YoIR1 distance, 4.9%; CMJ_h_, 3.8%; absolute PPO, 2.5%; relative PPO, 2.9%; absolute and relative PF, 3.8%. Customized spreadsheets and SPSS statistical software (version 24.0, IBM SPSS Statistics, Chicago, IL, USA) were utilised to perform data analysis.

## Results

Anthropometric characteristics and physical test results at T1, T2 and T3 and relative ANOVA outcomes are presented in Tables 1, 2 and 3. As four Division II and five Division III players did not perform the Yo-YoIR1 at all-time points during the season, their data were not included in the statistical analysis of this test.

**Table 1:** Anthropometric characteristics of division I, II and III players measured across the basketball season at various time points (i.e. before and after the preparation period and during the competitive season). The two-way repeated-measures ANOVA (Divisions × time) outcomes are presented; when significant *P* values were obtained, Bonferroni post hoc results were included.

		T1	T2	T3	ANOVA interaction *P* value (ƞ^2^)	ANOVA main effect time *P* value (ƞ^2^)	ANOVA main effect division *P* value (ƞ^2^)
Body mass (kg)	DIV I	99.3 ± 11.4	99.0 ± 11.1	98.5 ± 11.3	0.456 (0.050)	0.624 (0.013)	0.038 (0.171)
	DIV II	92.7 ± 12.7	92.4 ± 12.1	92.1 ± 11.9			
	DIV III	86.6 ± 11.7	86.4 ± 11.3	87.1 ± 11.8			
Body fat (%)	DIV I	13.3 ± 4.1	12.3 ± 4.1	12.1 ± 3.7	0.071 (0.121)	0.001 (0.203)	0.168 (0.097)
	DIV II	10.5 ± 3.2	10.4 ± 3.1	10.7 ± 3.3		T1 > (T2 = T3)	
	DIV III	10.8 ± 4.2	9.5 ± 3.7	9.5 ± 3.5			

Abbreviations: DIV, division; T1, before the preparation period; T2, after the preparation period; T3, during the competitive phase of the season.

**Table 2:** Running tests data of division I, II and III players measured across the basketball season at various time points (i.e. before and after the preparation period and during the competitive season). The two-way repeated-measures ANOVA (Divisions × time) outcomes are presented; when significant *P* values were obtained, Bonferroni post hoc results were included.

		T1	T2	T3	ANOVA interaction *P* value (ƞ^2^)	ANOVA main effect time *P* value (ƞ^2^)	ANOVA main effect division *P* value (ƞ^2^)
*Mognoni’s Test*							
MOG_[La-]_ (mmol∙L^-1^)	DIV I	4.3 ± 1.6	3.4 ± 1.3	3.4 ± 1.2	0.285 (0.069)	<0.001 (0.424)	0.396 (0.052)
	DIV II	4.8 ± 1.5	4.3 ± 1.4	3.4 ± 0.8		T1 > T2 > T3	
	DIV III	4.2 ± 1.5	3.5 ± 1.1	3.0 ± 1.0			
MOG_HR_ (bpm)	DIV I	165 ± 7	156 ± 6	159 ± 9	0.300 (0.066)	<0.001 (0.395)	0.655 (0.024)
	DIV II	168 ± 11	157 ± 6	160 ± 7		T1 > (T2 = T3)	
	DIV III	166 ± 11	162 ± 12	160 ± 10			
*High-intensity Intermittent Test*							
HIT_[La-]_ (mmol∙L^-1^)	DIV I	5.3 ± 2.6	3.9 ± 1.4	3.3 ± 1.5	0.062 (0.124)	<0.001 (0.652)	0.017 (0.208)
	DIV II	8.2 ± 2.5	4.8 ± 2.4	5.1 ± 1.5		T1 > (T2 = T3)	DIV I < DIV II
	DIV III	7.5 ± 1.7	4.9 ± 1.1	4.6 ± 1.5			
HIT_[H+]_ (mmol∙L^-1^)	DIV I	46.7 ± 6.7	43.2 ± 2.9	40.9 ± 2.4	0.146 (0.091)	<0.001 (0.495)	0.001 (0.313)
	DIV II	53.1 ± 6.5	45.5 ± 4.5	47.7 ± 4.0		T1 > (T2 = T3)	DIV I < (DIV II = DIV III)
	DIV III	52.8 ± 6.3	47.8 ± 3.7	47.2 ± 4.7			
HIT_[HCO3-]_ (mmol∙L^-1^)	DIV I	20.3 ± 3.5	21.9 ± 1.7	23.1 ± 2.4	0.106 (0.102)	<0.001 (0.572)	0.007 (0.250)
	DIV II	16.6 ± 2.4	20.4 ± 2.5	20.1 ± 2.1		T1 < (T2 = T3)	DIV I > DIV II
	DIV III	17.0 ± 3.3	20.8 ± 1.7	20.7 ± 2.7			
HIT_HR_ (bpm)	DIV I	160 ± 8	150 ± 5	149 ± 9	0.441 (0.051)	<0.001 (0.519)	0.064 (0.145)
	DIV II	164 ± 7	150 ± 8	155 ± 9		T1 > (T2 = T3)	
	DIV III	169 ± 12	156 ± 12	154 ± 11			
*Yo-Yo Intermittent Recovery Test—level 1*							
Distance (m)	DIV I	-	-	-			
	DIV II	1765 ± 324 *	2250 ± 247	2225 ± 217	0.035 (0.213)	<0.001 (0.789)	0.823 (0.004)
	DIV III	1610 ± 330 *	2140 ± 373	2390 ± 419		(T3 = T2) > T1	

Abbreviations: DIV, division; MOG, Mognoni’s test; HIT, High-intensity Intermittent Test; HR, heart rate; [H+], blood hydrogen ions concentration; [HCO3-], blood bicarbonates concentration; [La-], blood lactate concentration; T1, before the preparation period; T2, after the preparation period; T3, during the competitive phase of the season.

*, significantly lower than T2 and T3.

**Table 3:** Counter-movement jumping test data of division I, II and III players measured across the basketball season at various time points (i.e. before and after the preparation period and during the competitive season). The two-way repeated-measures ANOVA (Divisions × time) outcomes are presented; when significant *P* values were obtained, Bonferroni post hoc results were included.

		T1	T2	T3	ANOVA interaction *P* value (ƞ^2^)	ANOVA main effect time *P* value (ƞ^2^)	ANOVA main effect division *P* value (ƞ^2^)
CMJ_h_ (cm)	DIV I	46.9 ± 4.4	46.1 ± 5.6	47.2 ± 5.6	0.232 (0.076)	0.204 (0.044)	0.075 (0.138)
	DIV II	50.9 ± 5.6	49.7 ± 4.6	50.4 ± 4.4			
	DIV III	50.1 ± 4.8	51.1 ± 5.3	51.6 ± 5.1			
PPO (W∙kg^-1^)	DIV I	53.5 ± 4.8	55.3 ± 5.8	57.2 ± 5.1 #	0.037 (0.134)	<0.001 (0.231)	0.917 (0.005)
	DIV II	56.1 ± 5.2	56.1 ± 4.9	56.1 ± 4.8		T3 > T1	
	DIV III	54.4 ± 5.1	56.2 ± 5.8	57.2 ± 5.7			
PF (N∙kg^-1^)	DIV I	25.7 ± 1.9	26.9 ± 2.3	27.1 ± 2.3	0.376 (0.058)	0.019 (0.107)	0.368 (0.056)
	DIV II	25.9 ± 2.0	26.2 ± 3.0	26.1 ± 2.9			
	DIV III	25.1 ± 1.9	25.5 ± 2.4	25.6 ± 1.8			
PPO (W)	DIV I	5282 ± 582	5445 ± 562	5611 ± 681	0.065 (0.117)	0.001 (0.181)	0.072 (0.140)
	DIV II	5182 ± 745	5172 ± 722	5162 ± 732		T3 > T1	
	DIV III	4691 ± 624	4836 ± 680	4972 ± 783			
PF (N)	DIV I	2539 ± 271	2658 ± 345	2663 ± 348	0.354 (0.060)	0.051 (0.082)	0.002 (0.290)
	DIV II	2388 ± 294	2408 ± 318	2392 ± 332			DIV I > DIV III
	DIV III	2166 ± 249	2191 ± 285	2219 ± 282			

Abbreviations: CMJ_h_, Counter-movement jump height; DIV, division; PPO, peak power output; PF, peak force; T1, before the preparation period; T2, after the preparation period; T3, during the competitive phase of the season.

^#^, significantly higher than T1 within the division.

Across the monitored period no significant changes were observed for body mass among the divisions (*F*(2,70) = 0.475, *P* = 0.624, ƞ^2^ = 0.013), while body fat was significantly but minimally reduced after the preparation period (main effect of time: *F*(1.836,57.595) = 8.906, *P* = 0.001, ƞ^2^ = 0.203). A main effect of division was observed for body mass (*F*(2,35) = 3.607, *P*<0.038, ƞ^2^ = 0.171).

Significant moderate differences were found in physiological responses to Mognoni’s test (main effect of time: MOG_[La-]_, *F*(1.629,57.012) = 25.733, *P*<0.001, ƞ^2^ = 0.424; MOG_HR_, *F*(2,70) = 22.886, *P*<0.001, ƞ^2^ = 0.395). MOG_[La-]_ (*P*<0.001, change in mean = -15.70±13.81%, ES = -0.44±0.42) and MOG_HR_ (*P*<0.001, change in mean = -4.85±2.48%, ES = -0.82±0.43) were significantly reduced after the preparation period, while a further reduction was observed only for MOG_[La-]_ at T3 compared to T2 (*P* = 0.010, change in mean = -11.98±13.29%, ES = -0.37±0.40). There was a main effect of time for physiological responses to HIT (HIT_[La-]_, *F*(1.719,60.163) = 65.475, *P*<0.001, ƞ^2^ = 0.652; HIT_[H+]_, *F*(2,70) = 34.318, *P*<0.001, ƞ^2^ = 0.495; HIT_[HCO3-]_, *F*(2,70) = 46.816, *P*<0.001, ƞ^2^ = 0.572; HIT_[HR]_, *F*(2,70) = 37.819, *P*<0.001, ƞ^2^ = 0.519), with post-hoc analysis revealing improved physiological responses from T1 to T2 (HIT_[La-]_, *P*<0.001, change in mean = -33.61±13.45%, ES = -0.93±0.38; HIT_[H+]_, *P*<0.001, change in mean = -9.95±4.79%, ES = -0.74±0.37; HIT_[HCO3-]_, *P*<0.001, change in mean = 18.34±8.31%, ES = 0.85±0.37; HIT_[HR]_, *P*<0.001, change in mean = -7.58±2.55%, ES = -1.28±0.44). Furthermore, a main effect of division was observed for HIT_[La-]_ (*F*(2,35) = 4.588, *P* = 0.017, ƞ^2^ = 0.208), HIT_[H+]_ (*F*(2,35) = 7.972, *P* = 0.001, ƞ^2^ = 0.313) and HIT_[HCO3-]_ (*F*(2,35) = 5.824, *P* = 0.007, ƞ^2^ = 0.250), with Division I players showing better physiological responses compared to Division II (i.e. HIT_[La-]_, *P* = 0.023 change in mean = -32.93±15.82%, ES = -0.69±0.41; HIT_[HCO3-]_, vs Division II: *P* = 0.010 change in mean = 14.68±7.71%, ES = 0.93±0.45) or both Division II and Division III counterparts (i.e. HIT_[H+]_, vs Division II: *P* = 0.008, change in mean = -10.54±4.63%, ES = -0.86±0.42 and vs Division III: *P* = 0.003, change in mean = -11.50±4.25%, ES = -1.01±0.42). As regards Yo-YoIR1 performance, the two-way ANOVA showed significant interaction between divisions across the selected time points (*F*(2,28) = 3.792, *P* = 0.035, ƞ^2^ = 0.213). Yo-YoIR1 performance was significantly increased from T1 to T2 in both Division II (*P*<0.001 change in mean = 28.62±21.08%, ES = 1.33±0.85) and III (*P*<0.001 change in mean = 33.45±26.45%, ES = 1.43±1.02).

A statistically significant interaction was observed for relative PPO during CMJs (*F*(4,70) = 2.709; *P* = 0.037, ƞ^2^ = 0.134). Post hoc analysis revealed relative PPO produced at T3 by Division I players to be higher compared to T1 (*P*<0.001, change in mean = 6.97±7.55%, ES = 0.73±0.78). Furthermore, a small effect of time was found for absolute PPO (*F*(2,70) = 7.730, *P* = 0.001, ƞ^2^ = 0.181; post hoc: T3vsT1, *P*<0.001, change in mean = 3.87±6.91%, ES = 0.29±0.47), relative PPO (*F*(2,70) = 10.529, *P*<0.001, ƞ^2^ = 0.231; post hoc: T3vsT1, *P*<0.001, change in mean = 4.14±4.32%, ES = 0.44±0.45) and relative PF (*F*(2,70) = 4.174, *P* = 0.019, ƞ^2^ = 107) produced during CMJs. A greater absolute PF was produced during CMJs by Division I than Division III players (main effect of division, *F*(2,35) = 7.154, *P*<0.002, ƞ^2^ = 0.290, post hoc: *P* = 0.002, change in mean = 19.51±6.67%, ES = 1.58±0.49).

From the analysis of individual responses (Table 4), we observed that 71% of the players involved in the study did not show a very likely reduction in their body fat percentage from T1 to T2. Furthermore, 21% of the players showed a very likely change in body fat from T2 to T3. Contrastingly to the significant improvement observed from T1 to T2 and from T2 to T3 in MOG_[La-]_, the individual analysis showed that 55% and 66% of players did not very likely reduce MOG_[La-]_ in these periods respectively. Regarding YoYoIR1 and HIT, 40% and 34% of the players respectively did not very likely improved their results during the preparation period, while 16% and 34% displayed very likely changes from T2 to T3. Only few players for each division showed very likely variations in CMJ parameters across the different phases of the season.

**Table 4:** Within-subject very likely changes in anthropometric characteristics and physical test outcomes between the three testing sessions (performed before and after the preparation period and during the competitive season) according to the competitive level of play (i.e. division I, II and III).

		T2 vs T1	T3 vs T2	T3 vs T1
*DIVISION I*				
*Anthropometrics*	Body mass (kg)	<1		<2
	Body fat (%)	<4	2 (<1; >1)	<6
*Mognoni’s Test*	MOG_[La-]_ (mmol∙L^-1^)	8 (<7; >1)	4 (<2; >2)	<7
	MOG_HR_ (bpm)	<9	6 (<2; >4)	9 (<8; >1)
*HIT Test*	HIT_[La-]_ (mmol∙L^-1^)	8 (<6; >2)	7 (<6; >1)	9 (<8; >1)
	HIT_[H+]_ (mmol∙L^-1^)	<5	<1	<6
	HIT_[HCO3-]_ (mmol∙L^-1^)	>5		>6
	HIT_HR_ (bpm)	<8	7 (<4; >3)	<7
*Yo-YoIR1 Test*	Distance (m)	-	-	-
*CMJ test*	CMJ_h_ (cm)	<2	>2	<1
	PPO (W∙kg^-1^)	>2	>3	>4
	PF (N∙kg^-1^)	>4	>1	>5
	PPO (W)	>3	>3	>3
	PF (N)	>3	>1	>2
*DIVISION II*				
*Anthropometrics*	Body mass (kg)		>1	2 (<1; >1)
	Body fat (%)	4 (<2; >2)	4 (<2; >2)	5 (<2; >3)
*Mognoni’s Test*	MOG_[La-]_ (mmol∙L^-1^)	6 (<4; >2)	<7	<8
	MOG_HR_ (bpm)	<8	>4	9 (<8; >1)
*HIT Test*	HIT_[La-]_ (mmol∙L^-1^)	<9	3 (<1; >2)	<10
	HIT_[H+]_ (mmol∙L^-1^)	<7	>2	<6
	HIT_[HCO3-]_ (mmol∙L^-1^)	>7	3 (<2; >1)	>7
	HIT_HR_ (bpm)	<9	>4	<6
*Yo-YoIR1 Test*	Distance (m)	>7		>7
*CMJ test*	CMJ_h_ (cm)	<2		3 (<2; >1)
	PPO (W∙kg^-1^)		<1	2 (<1; >1)
	PF (N∙kg^-1^)	3 (<1; >2)	4 (<2; >2)	2 (<1; >1)
	PPO (W)		2 (<1; >1)	<1
	PF (N)	3 (<1; >2)	4 (<2; >2)	2 (<1; >1)
*DIVISION III*				
*Anthropometrics*	Body mass (kg)	<1	>1	>1
	Body fat (%)	<5	>2	9 (<7; >2)
*Mognoni’s Test*	MOG_[La-]_ (mmol∙L^-1^)	6 (<5; >1)	<4	<9
	MOG_HR_ (bpm)	8 (<5; >3)	8 (<5; >3)	<6
*HIT Test*	HIT_[La-]_ (mmol∙L^-1^)	<9	<3	<11
	HIT_[H+]_ (mmol∙L^-1^)	<4	2 (<1; >1)	<7
	HIT_[HCO3-]_ (mmol∙L^-1^)	>7		>7
	HIT_HR_ (bpm)	9 (<8; >1)	4 (<2; >2)	<8
*Yo-YoIR1 Test*	Distance (m)	>8	>4	>7
*CMJ test*	CMJ_h_ (cm)	>1	>1	>1
	PPO (W∙kg^-1^)	>3	>1	4 (<1; >3)
	PF (N∙kg^-1^)	2 (<1; >1)	>1	<1
	PPO (W)	>3	>2	5 (<1; >4)
	PF (N)	2 (<1; >1)	>1	2 (<1; >1)

For each variable, the number of subjects who showed a very likely change (i.e. probability of a positive or negative change >90%) is shown. When individual changes were found in opposite directions, the number of subjects increasing (>) or decreasing (<) values is reported in parentheses. CMJ_h_, Counter-movement jump height; MOG, Mognoni’s test; HIT, High-intensity Intermittent Test; HR, heart rate; PPO, peak power output; PF, peak force; Yo-YoIR1, Yo-Yo Intermittent Recovery Test; [H+], blood hydrogen ions concentration; [HCO3-], blood bicarbonates concentration; [La-], blood lactate concentration; T1, before the preparation period; T2, after the preparation period; T3, during the competitive phase of the season.

## Discussion

The present study provides novel insights pertinent to physical capacities of basketball players selected from various teams and playing standards (i.e. elite to semi-professional). Unique to previous studies, the current investigation provides relevant data spanning an entire season including both the preparation and competitive phases. The main improvements in physical capacities occurred during the preparation period, when the aerobic fitness and the ability to sustain high-intensity intermittent efforts were moderately-to-largely improved among all Divisions. Furthermore, no significant or only small changes in CMJ variables were reported across the different periods. Overall, Division I players are characterized by a better ability to perform high-intensity intermittent exercises than lower division players.

The present results indicate that anthropometric characteristics of basketball players are minimally affected by the different phases of the season. Athletes’ body mass was maintained at all-time points among the Divisions, while a small reduction in body fat percentage was reported post the preparation period. Similar results were previously reported among NCAA and NBA basketball players [8, 10, 12, 13]. However, it is noteworthy that at individual level only 6 Division I, 2 Division II and 2 Division III players demonstrated a very likely reduction in their body fat percentage from T1 to T3, while 3 Division II and 2 Division III players increased it. This may be due to the different specific workout performed and diet followed by the players across the entire season to achieve specific individual anthropometric goals (e.g. increase muscle mass, reduce body fat, gain body mass).

According to previous investigations [6, 21, 25], the physiological responses to a submaximal continuous running test (Mognoni’s test) were used to evaluate the aerobic fitness of basketball and soccer players. The effectiveness of the preparation period to enhance the aerobic fitness among basketball players was confirmed by the moderately improved physiological responses to the Mognoni’s test (i.e. MOG_[La-]_ and MOG_HR_) measured at T2 compared to T1. Additionally, blood lactate concentrations measured after the test were further reduced during the in-season phase, indicating that the competitive basketball period may have positively affected the aerobic capacities of players. Contrastingly to MOG_[La-]_, MOG_HR_ remained stable from T2 to T3; however, this may be due to the analysis of HR responses in absolute terms (bpm) and not in percentage (%) to the maximal HR. Indeed, a prolonged period of training at high physiological loading during the different seasonal phases might have affected the maximal HR [35]. Furthermore, it has been reported that HR showed some limitations as indicator of aerobic performance capacity [36]. From the analysis of individual responses, it should be noted that MOG_[La-]_ was reduced after the preparation period among 8 Division I players (i.e. 53%), while at lower level only ~35% of players reported improved physiological responses to the test. This underlines a greater efficacy of the strategies adopted by the Division I coaching staff or, a greater ability of Division I players to enhance their aerobic capacities during the preparation period. Overall these findings confirm previous studies reporting aerobic fitness capacity to be improved following the preparation period and to be preserved or slightly increased during the competitive phase of the season [6, 9–11, 13–16]. Accordingly, the development of the aerobic capacities before the commencement of the competitive season may positively assist the reoxygenation of myoglobin and resynthesis of phosphoryl-creatine during basketball activities [37, 38]. In conclusion, despite the low ecological validity and the low-level of specificity of this test due to the intermittent nature of basketball games, the results of the present study confirm that the Mognoni’s test can be efficiently and practically used to monitor the aerobic adaptations of basketball athletes, especially at professional level where it may be difficult to perform maximal tests during the competitive season.

Several studies have analysed seasonal changes in Yo-YoIR1 performance among adult athletes in different team sports like soccer [39], but only few studies have investigated basketball [6, 16]. The Yo-YoIR1 performance was significantly improved (~20–30%) after the preparation period in both Division II and III groups, thus confirming previous results reported in professional adult basketball players [6, 16]. However, no further improvements in Yo-YoIR1 performance were recorded from T2 to T3. It should be noted that 40% of the players involved in the study did not (very likely) improved their YoYoIR1 performance during the preparation period, while 16% displayed very likely changes from T2 to T3. Thus, the relevant number of players showing contradictory results suggests the need to use an individualized approach when monitoring and prescribing workloads across these periods. As such, training responses may be affected by different workloads [6], while players can show different exercise-induced adaptations at individual level [40]. During the competitive phase of the season, the Yo-YoIR1 performance of Division II and Division III was lower compared to Tunisian National players (2619 ±731 m) [41], but similar to previously reported data for athletes competing in the same Italian tournaments [21]. Overall YoYoIR1 appears to be effective in monitoring seasonal changes in the ability to perform maximal high-intensity efforts, however, practitioners should consider the difficulties associated with the use of a maximal test with professional athletes during the competitive phase of the season. Indeed, Division I players of the present study did not performed the Yo-YoIR1 due to restrictions made by technical coaches, while four Division II and five Division III players were not able to carry out the test at all-time points during the season.

The ability to sustain high-intensity intermittent exercises, evaluated measuring the physiological responses to HIT, has been shown to be a key component to discriminate players of different competitive level [21]. In the present study the physiological responses to HIT were influenced by the different phases of the season. Similar to YoYoIR1 performance, during the preparation period the players moderately-to-largely developed their ability to sustain a submaximal high-intensity intermittent exercise, while this ability was preserved during the competitive phase of the season. It should be noted that following the preparation period, less than 50% of Division I players (n = 6), but more than ~70% of Division II (n = 9) and Division III (n = 9) players, very likely reduced HIT_[La-]_. This difference might be attributed to the greater fitness status of higher-level players before the commencement of the preparation period, likely as a consequence of the greater detraining occurred during the off-season among lower-level players. Overall, post hoc analysis revealed no further statistical improvements in HIT from T2 to T3 among the divisions. However, interestingly 6 Division I players further reduced HIT_[La-]_ during the competitive phase of the season (i.e. T3 vs T2). Thus, suggesting a further enhancement of their ability to maintain acid-base balance during submaximal intermittent exercise, reducing the anaerobic contribution to the test and improving the buffering capacity [42]. The additional adaptations observed among these Division I players during the competitive phase of the season may be a consequence of the greater intermittent workload and high-intensity phases exerted by elite players during basketball games [1, 43]. In addition, higher-level competitive players usually undergo a greater training load compared to their lower level counterparts [5]. Training load is moderately associated with beneficial variation in physiological responses (i.e. HIT_[La-]_, HIT_[H+]_) to HIT [6]. Accordingly, the results of the current study confirm that Division I players are characterized by better physiological responses (i.e. HIT_[La-]_, HIT_[H+]_, HIT_[HC03-]_) when sustaining high-intensity intermittent exercise compared to lower division counterparts. Overall, these results support previous findings [6], suggesting that the measurement of physiological responses to a submaximal high-intensity intermittent exercise could represent a valid approach to investigate the training adaptations across an entire basketball season.

Studies comparing changes in strength capacities of basketball players across different seasonal phases reported contrasting results [4, 9–15]. In the present study, a significant main effect of time was observed for PPO (in both absolute and relative terms) and relative PF produced during CMJs, with post hoc analysis revealing lower PPO values in T1. However, it should be considered that all the observed changes were small, while from the analysis of individual responses, only few players for each division reported significant variations in CMJ parameters across the different phases of the season. Overall, it appears that the preparation period does not impact consistently on CMJ variables [5]. A possible explanation for this is that the players completed an ineffective training stimuli, or an overreaching phenomenon occurred during this phase [44]. Accordingly, the use of high workload can negatively affect PPO production during CMJs and neuromuscular properties [5]. Otherwise, relative and absolute PPO during CMJs were slightly increased during the competitive phase of the season (i.e. T1 vs T3) likely as a consequence of the occurred optimization of neuromuscular properties [5, 7, 44].

There are some limitations that should be considered from this research. Athletes were selected from just one national tournament; thus, data collection might not be representative of overall basketball playing population. In addition, only a limited number of anthropometric and physiological capacities could be assessed. To develop a more holistic understanding of these capacities among European basketball players, we suggest that future studies utilize a wider range of test parameters. For example, this study does not provide any information about agility and about the ability to accelerate, decelerate and change direction. Furthermore, due to the difficulties in assessing professional players, the evaluations have not been performed at the end of the competitive season (i.e. June) and before the commencement of the subsequent one. Thus, the present results do not provide detailed information about detraining during the off-season. The recruited teams were located around the Italy and technical coaches agreed to perform the testing sessions at the start of the week if the official games were scheduled on Saturday or Sunday. Furthermore, to avoid potential confounding effects of prior exercise fatigue on the outcome variables the testing sessions were performed after a day without training (i.e. day off). As such, in-season data were collected over a 6-weeks period due to logistical and restrictions issues.

### Practical applications

The present study provides practitioners with applied physical capacity knowledge pertinent in adult basketballers across seasonal phases. An individual approach should be considered when interpreting the changes in physical capacities of basketball teams. Indeed, each athlete undergoes different training and game loads which might lead to different physical adaptations. For example, contrastingly to the significant improvement observed across the preparation and competitive periods in MOG_[La-]_, the individual analysis showed that more than half of players involved in the study did not (very likely) reduce MOG_[La-]_ in these periods. Furthermore, despite HIT_[La-]_ was considerably reduced after the preparation period, the analysis of individual responses showed HIT_[La-]_ to be decreased only among 6 Division I players, remaining stable or increasing in 5 and 2 Division I players respectively. As such, we recommend physical tests be used to evaluate selected fitness capacities and to monitor fatigue status of players during the different seasonal phases of basketball. Consequently, strength and conditioning coaches should focus on developing individualized training programs based on areas of athlete weakness, and on assisting technical coaches in the development of the weekly training schedule of the team. The development of specific adjustments to the player’s daily routine (e.g. recovery intervention or additional specific trainings) might represent a useful strategy to enhance athlete’s performance. Accordingly, the results of the present study assist basketball practitioners of different levels in the interpretation of physical fitness changes across the season. Data of this study should be used to better interpret the fitness status of the player during the season, when no previous physical assessment information are available (e.g. new signed players).

## Supporting information

S1 File Anthropometric, physical and physiological data. (XLSX)
